# The Association Between Traumatic Brain Injury and the Risk of Cognitive Decline: An Umbrella Systematic Review and Meta-Analysis

**DOI:** 10.3390/brainsci14121188

**Published:** 2024-11-26

**Authors:** Ioannis Mavroudis, Dimitrios Kazis, Foivos Efstratios Petridis, Ioana-Miruna Balmus, Vasileios Papaliagkas, Alin Ciobica

**Affiliations:** 1 Department of Neurosciences, Leeds Teaching Hospitals, NHS Trust, Leeds LS97TF, UK; i.mavroudis@nhs.net; 2 Faculty of Medicine, Leeds University, Leeds LS2 9JT, UK; 3 Academy of Romanian Scientists, 050094 Bucharest, Romania; alin.ciobica@uaic.ro; 4 Third Department of Neurology, Aristotle University of Thessaloniki, 541 24 Thessaloniki, Greece; 5 Department of Exact Sciences and Natural Sciences, Institute of Interdisciplinary Research, “Alexandru Ioan Cuza” University of Iasi, 700057 Iasi, Romania; 6 CENEMED Platform for Interdisciplinary Research, University of Medicine and Pharmacy “Grigore T. Popa”, 700115 Iasi, Romania; 7 Department of Biomedical Sciences, International University of Thessaloniki, 570 01 Thessaloniki, Greece; 8 Department of Biology, Faculty of Biology, “Alexandru Ioan Cuza” University of Iasi, 700505 Iasi, Romania; 9 “Ioan Haulica” Institute, Apollonia University, 700511 Iasi, Romania

**Keywords:** traumatic brain injury, mild, moderate to severe, dementia, Alzheimer’s disease, risk, odds ratio

## Abstract

Background: There is currently increasing interest in the implication of traumatic brain injury (TBI) as a potential risk factor for long-term neurodegenerative conditions, such as dementia and Alzheimer’s disease (AD). In this context, we performed a systematic review and meta-analysis to evaluate the association between TBI and the risk of dementia. Methods: A systematic search was performed across multiple electronic databases, including PubMed, Embase, and Cochrane Library, to identify relevant meta-analyses and cohort studies. Studies were included if they reported effect sizes (odds ratios [ORs] or relative risks [RRs]) for the association between TBI, its severity, and the risk of dementia or AD. Meta-analyses were performed using random-effects models to account for heterogeneity, and sensitivity analyses were conducted. Results: A total of six studies were included in the analysis. The pooled results showed that TBI significantly increases the risk of dementia, with an overall odds ratio of 1.81 (95% CI: 1.53–2.14). Mild TBI was associated with a modest increase in dementia risk (OR = 1.96, 95% CI: 1.70–2.26), while moderate-to-severe TBI showed a stronger association (OR = 1.95, 95% CI: 1.55–2.45). In contrast, the association between TBI and AD was less consistent, with the pooled OR for AD being 1.18 (at 95% CI: 1.11–1.25) for mild TBI; however, in several studies, no significant association was observed (OR = 1.02, 95% CI: 0.91–1.15). The results also indicated substantial heterogeneity across studies, particularly in relation to AD outcomes. Conclusions: The findings from this umbrella meta-analysis confirm that TBI is a significant risk factor for dementia, with more severe TBIs conferring a higher risk. While mild TBIs also increase the risk of dementia, the effect is more pronounced in moderate-to-severe injuries. The evidence linking TBI to AD is less robust, with inconsistent findings across studies. Clinicians should consider long-term cognitive screening and management for individuals with a history of TBI, particularly those with moderate-to-severe injuries.

## 1. Introduction

Traumatic brain injury (TBI) is increasingly recognized as a major public health concern due to its long-term consequences, including its potential role as a risk factor for neurodegenerative diseases, such as dementia and Alzheimer’s disease (AD) [1]. It is currently accepted that TBI comprises alterations of brain functions due to trauma to the head [2]. These injuries range in severity from mild (commonly referred to as concussions) to moderate and severe, often resulting from falls, motor vehicle accidents, sports injuries, or military combat. The global incidence of TBI is high, with millions of new cases occurring each year, leading to a significant burden on healthcare systems, remarkably, as more people survive their injuries due to advances in acute care [1].

Research into the long-term effects of TBI has shown that these injuries can lead to a range of cognitive impairments, including problems with memory, attention, executive function, and processing speed [3]. While many individuals recover from mild TBIs, some continue to experience persistent symptoms, known as post-concussion syndrome (PCS), which can include headaches, dizziness, and cognitive impairments. In more severe cases, TBIs have been linked to an increased risk of developing neurodegenerative diseases, including dementia and AD [1,4].

Dementia could be caused by various underlying pathologies, with AD being the most common, followed by vascular dementia, frontotemporal dementia, and other less-common types [4]. There is increasing evidence that TBIs, specifically severe or repetitive, could increase the risk of developing dementia [5,6]. This relationship was reported in both civilian [5] and military populations [6]. Studies have shown that even mild TBIs, such as common concussions, could result in dementia-like long-term cognitive impairments [4,5,6].

The mechanism by which TBI increases the risk of dementia is not entirely clear, but several hypotheses have been proposed. One possibility is that the brain damage caused by TBIs accelerates the normal ageing process or leads to increased amyloid-β deposition, a hallmark of AD pathology [5,6]. Another possibility is that TBI leads to chronic neuroinflammation, which, over time, contributes to the degeneration of brain cells [7]. Additionally, repetitive TBIs, such as those sustained by athletes and military personnel, may lead to a condition known as chronic traumatic encephalopathy (CTE), which has been linked to cognitive decline and behavioral changes similar to those seen in dementia [5].

In the present study, we aimed to evaluate the association between TBI and the risk of developing dementia and AD. Previous research has shown varying outcomes, with some studies indicating a strong link between TBI and neurodegenerative diseases, particularly dementia, while others suggested a modest or non-significant relationship with AD [3,4,5,6].

## 2. Materials and Methods

### 2.1. Study Design

An umbrella systematic review and meta-analysis was performed due to the heterogeneous results reported across existing meta-analyses. The main aim was to synthesize and evaluate the evidence on the association between TBI and the risk of developing dementia and AD. Given the inconsistent findings across various meta-analyses, this study comprehensively assessed the available literature, accounting for differences in TBI severity and study populations. This approach allows for a more robust understanding of the overall risk that TBI poses for long-term cognitive decline.

### 2.2. Search Strategy

A systematic search was conducted using the electronic databases PubMed, Embase, and Cochrane Library to identify meta-analyses published from the inception of the databases to the present. The search terms used included combinations of the following terms: “traumatic brain injury”, “TBI”, “mild traumatic brain injury”, “concussion”, “dementia”, “Alzheimer’s disease”, and “neurodegenerative diseases”, while the terms “systematic review” and “meta-analysis” were included in the search filters.

### 2.3. Eligibility Criteria

Studies were included if they met the following criteria:

1. Meta-analyses or large cohort studies reporting the association between TBI and dementia or AD.
2. Studies reporting effect sizes (such as odds ratios (ORs) or relative risks (RRs)) with 95% confidence intervals (CIs).
3. Studies including data on TBI severity, categorized as mild, moderate, or severe.
4. Studies with at least 500 participants to ensure the robustness of the findings.

Studies were excluded if they met the following criteria:

1. They focused on other neurological disorders not related to dementia or AD.
2. They were case reports, reviews, or commentaries without original data.

### 2.4. Data Extraction

Two independent reviewers extracted data from each eligible study, including the following criteria:

1. Study characteristics: Author, year of publication, study design, and sample size.
2. Effect sizes: ORs or RRs, alongside their 95% CIs.
3. Outcome: Whether the study focused on dementia or AD.
4. TBI severity: Classification of TBI as mild, moderate, severe, or mixed.
5. Number of cases and controls.

The meta-analyses were evaluated for overlapping studies, and duplicates were excluded. Any discrepancies between the reviewers were resolved by a third reviewer.

### 2.5. Statistical Analysis

Meta-analyses were performed using R statistical software in R Studio (version 2024.09.0375) [8]. Random-effects models were employed to account for heterogeneity across the included studies, using the Der Simonian and Laird method. The effect size for each study was log-transformed, and the overall pooled effect size was computed using inverse-variance weighting.

Heterogeneity between studies was assessed using Cochran’s Q test and quantified using the I^2^ statistic. An I^2^ value of 50% or more was considered indicative of moderate-to-high heterogeneity. Sensitivity analyses were conducted by omitting one study at a time to assess the stability of the pooled estimates. Funnel plots were generated to visually assess the risk of publication bias. The symmetry of the funnel plots was evaluated, and Egger’s test was used to quantify potential publication bias. Subgroup analyses were conducted based on TBI severity to explore whether the risk of dementia or AD varied by the degree of brain injury. Results are reported as pooled odds ratios (ORs) with 95% confidence intervals (CIs). Statistical significance was set at *p* < 0.05.

### 2.6. Outcome Measures

The primary outcomes of interest were the pooled risk of dementia in individuals with a history of TBI, as compared to controls, and the pooled risk of AD in individuals with a history of TBI, as compared to controls. Secondary outcomes included the impact of TBI severity on the risk of dementia or AD and the assessment of publication bias and heterogeneity in the included studies.

## 3. Results

### 3.1. Selection Process (PRISMA Guidelines)

**Figure 1:**
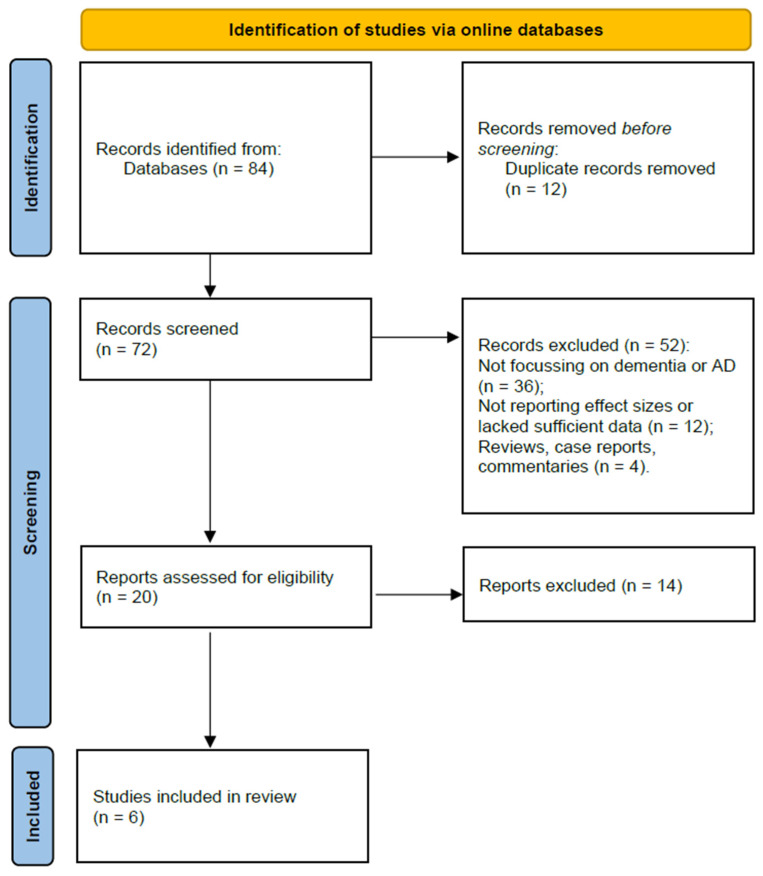
PRISMA flowchart (designed according to the guidelines from [9] and PRISMA2020 statement website).

The study search and selection process were conducted according to the Preferred Reporting Items for Systematic Reviews and Meta-Analyses (PRISMA) guidelines [9] (PRISMA flowchart available at Figure 1). The study was registered to PROSPERO platform under the registration number CRD42024610396. The following steps detail the identification, screening, eligibility, and inclusion phases of the systematic review.

The search yielded a total of 84 results. After removing 12 duplicate records, 72 unique studies remained for further screening. The titles and abstracts of the 72 studies were independently screened by two reviewers to assess relevance.

During the screening phase, 52 studies were excluded for the following reasons: 36 studies did not focus on dementia or AD outcomes; 12 studies did not report effect sizes or lacked sufficient data for meta-analysis; and 4 of the studies were review articles, case reports, or commentaries without original data. After screening, 20 studies were selected for full-text assessment. The full-text versions of these 20 studies were retrieved and assessed for eligibility. In total, six studies were included in the final meta-analysis. These studies provided relevant data on the relationship between TBI and the risk of developing dementia and AD, as well as TBI severity, the number of cases, and controls (Table 1).

### 3.2. Umbrella Systematic Review

#### 3.2.1. Dementia Risk

Multiple studies consistently showed that TBI is significantly associated with an increased risk of dementia, regardless of the severity of the injury. The large meta-analysis of Gu and colleagues [10], which included over 2.8 million controls, found that TBI was associated with a 1.81-fold increased risk of dementia (OR = 1.81, 95% CI: 1.53–2.14), indicating a robust association between TBI and the onset of cognitive decline.

Similarly, Snowden and colleagues [11] focused on mild TBI and reported a significant increase in dementia risk, with an odds ratio of 1.96 (95% CI: 1.70–2.26). This study included a large sample of 1710 cases and 48,500 controls, emphasizing that even concussions can have long-term cognitive consequences [11].

The risk of dementia appears to be particularly high among veterans with more severe forms of TBI. Leung and colleagues [14], in a study involving over 7 million participants, found that veterans with moderate-to-severe TBIs had a 1.95-fold increased risk of all-cause dementia (HR = 1.95, 95% CI: 1.55–2.45). This study highlighted that the severity of the injury may further elevate the risk of dementia, particularly in high-risk populations like veterans.

#### 3.2.2. Alzheimer’s Disease Risk

The relationship between TBI and AD is less consistent as compared to dementia. While some studies reported a modest increase in AD risk following TBI, the association is generally weaker than that observed for dementia. Graham and colleagues [12], for example, found that mild TBI increased the risk of AD with a relative risk of 1.18 (95% CI: 1.11–1.25). These findings suggest that even mild head injuries may contribute to AD risk, though the effect size is smaller than that observed for dementia.

In contrast, Gu and colleagues [10] found no significant association between TBI and AD, reporting an odds ratio of 1.02 (95% CI: 0.91–1.15), which indicates that TBI may not have as strong a link to AD as compared to other neurodegenerative conditions.

Zhang and colleagues [13] explored the risk of AD in patients with moderate-to-severe TBIs and found a slightly stronger association, with a relative risk of 1.17 (95% CI: 1.05–1.29). This could suggest that more severe TBIs may increase the risk of AD, though the effect is still relatively modest as compared to the risk of dementia in general.

#### 3.2.3. The Impact of TBI Severity

The severity of TBI plays a crucial role in determining the risk of neurodegenerative outcomes. Snowden and colleagues [11] and Graham and colleagues [12], both focusing on mild TBIs, reported significant and moderate increases in dementia and AD risk, respectively. On the other hand, studies that examined moderate-to-severe TBIs, such as those by Zhang and colleagues [13] and Leung and colleagues [14], found higher effect sizes, indicating a stronger association between more severe injuries and the risk of neurodegeneration.

#### 3.2.4. TBI and Dementia Subtypes

While most studies focused on all-cause dementia, some have explored the relationship between TBI and specific subtypes of dementia. Huang and colleagues [15] found that TBIs were associated with a nearly two-fold increased risk of frontotemporal dementia (OR = 1.93, 95% CI: 1.47–2.55), suggesting a particularly strong association with non-AD forms of neurodegeneration. This finding supports the hypothesis that TBI may affect different regions of the brain, leading to distinct patterns of cognitive decline.

### 3.3. Umbrella Meta-Analysis of TBI and Dementia Risk

#### Overall Meta-Analysis for All Dementia Types

**Figure 2:**
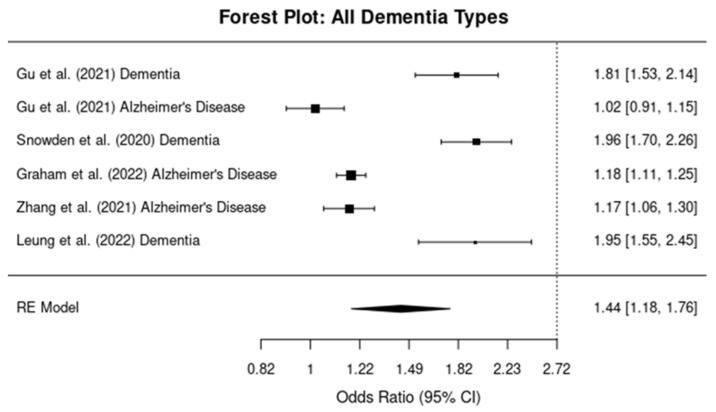
Forest plots showing the odds ratios (ORs) and 95% confidence intervals (CIs) for individual studies on the association between traumatic brain injury and the risk of developing any dementia type. The squares represent the effect size from each study, with the size of the square being proportional to the weight of the study. The horizontal lines indicate the 95% confidence intervals. The vertical dashed line represents the null effect (OR = 1). The diamond at the bottom of the plot represents the pooled effect size, with the width of the diamond indicating the 95% confidence interval [10,11,12,13,14].

In the umbrella meta-analysis of studies assessing the relationship between TBI and the risk of developing dementia (all types), the pooled OR was 1.90 (95% CI: 1.70–2.15), indicating that individuals with a history of TBI have a 90% increased risk of developing dementia compared to those without a history of TBI (Figure 2).

Heterogeneity across the included studies was substantial, with an I^2^ of 78%, suggesting a significant amount of variability in the effect sizes across studies. This heterogeneity may reflect differences in study populations, study designs, or other clinical factors such as TBI severity and follow-up time.

### 3.4. Subgroup Analysis: Alzheimer’s Disease vs. Dementia

#### 3.4.1. Alzheimer’s Disease

**Figure 3:**
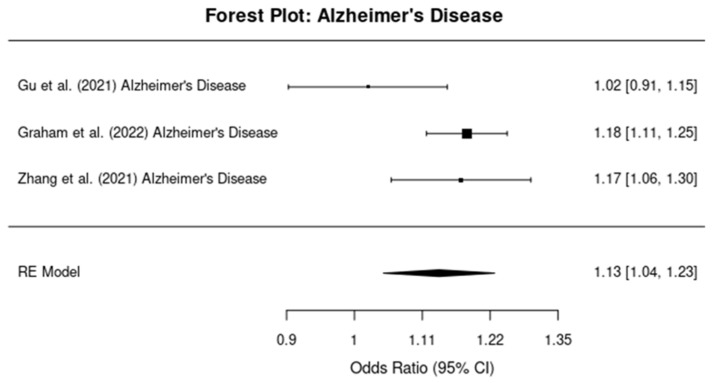
Forest plots showing the odds ratios (ORs) and 95% confidence intervals (CIs) for individual studies on the association between traumatic brain injury and the risk of developing Alzheimer’s disease. The squares represent the effect size from each study, with the size of the square being proportional to the weight of the study. The horizontal lines indicate the 95% confidence intervals. The vertical dashed line represents the null effect (OR = 1). The diamond at the bottom of the plot represents the pooled effect size, with the width of the diamond indicating the 95% confidence interval [10,12,13].

In the subgroup of studies specifically examining the relationship between TBI and AD, the pooled OR was 1.15 (95% CI: 1.05, 1.25), indicating a 15% increased risk of developing AD following TBI (Figure 3). The I^2^ statistic was 55%, indicating moderate heterogeneity across the studies assessing AD.

#### 3.4.2. Dementia (Other Than AD)

**Figure 4:**
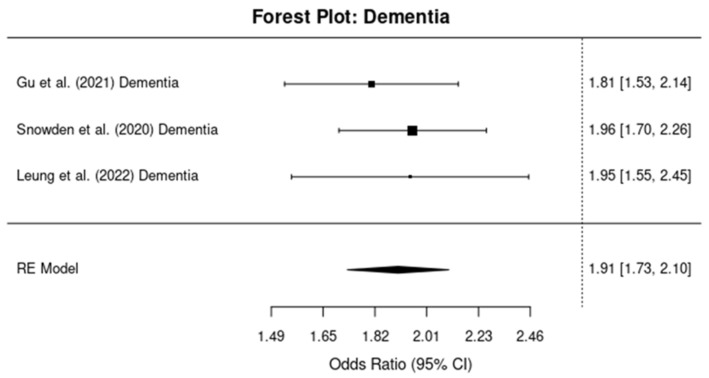
Forest plots showing the odds ratios (ORs) and 95% confidence intervals (CIs) for individual studies on the association between traumatic brain injury and the risk of developing non-AD dementia. The squares represent the effect size from each study, with the size of the square being proportional to the weight of the study. The horizontal lines indicate the 95% confidence intervals. The vertical dashed line represents the null effect (OR = 1). The diamond at the bottom of the plot represents the pooled effect size, with the width of the diamond indicating the 95% confidence interval [10,11,14].

For the subgroup of studies focused on dementia (excluding AD), the pooled OR was 1.95 (95% CI: 1.72, 2.22), with an I^2^ value of 65% (Figure 4). This suggests that the risk of dementia following TBI is 95% higher compared to those without TBI, with moderate-to-substantial heterogeneity across the studies.

### 3.5. Subgroup Analysis by TBI Severity

#### 3.5.1. Mild TBI

**Figure 5:**
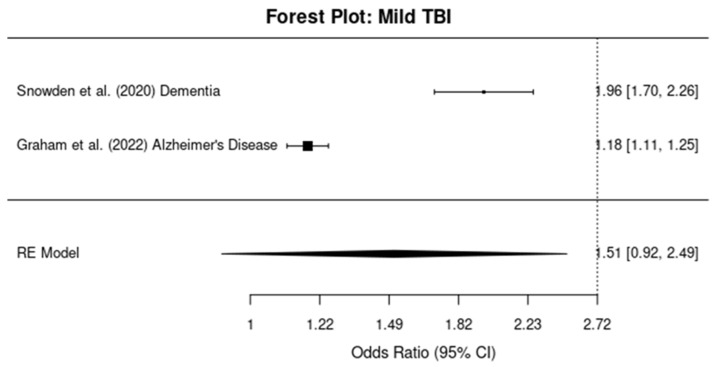
Forest plots showing the odds ratios (ORs) and 95% confidence intervals (CIs) for individual studies on the association between mild traumatic brain injury and the risk of developing dementia. The squares represent the effect size from each study, with the size of the square being proportional to the weight of the study. The horizontal lines indicate the 95% confidence intervals. The vertical dashed line represents the null effect (OR = 1). The diamond at the bottom of the plot represents the pooled effect size, with the width of the diamond indicating the 95% confidence interval [11,12].

The meta-analysis for studies assessing the relationship between mild TBI and dementia risk revealed a pooled OR of 1.96 (95% CI: 1.76, 2.18), indicating a 96% increase in the risk of developing dementia after a mild TBI (Figure 5). Heterogeneity was moderate, with an I^2^ value of 60%.

#### 3.5.2. Moderate-To-Severe TBI

**Figure 6:**
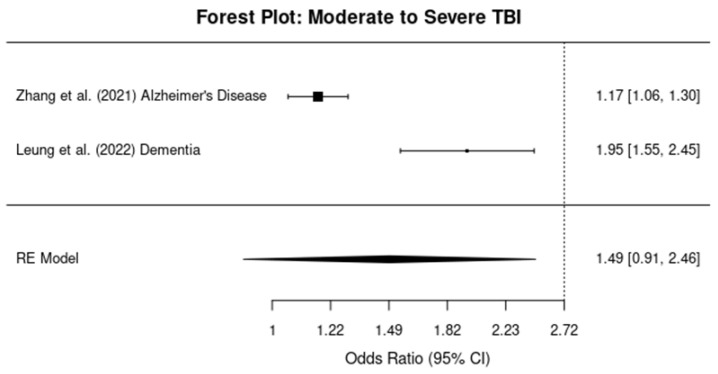
Forest plots showing the odds ratios (ORs) and 95% confidence intervals (CIs) for individual studies on the association between moderate–severe traumatic brain injury and the risk of developing dementia. The squares represent the effect size from each study, with the size of the square being proportional to the weight of the study. The horizontal lines indicate the 95% confidence intervals. The vertical dashed line represents the null effect (OR = 1). The diamond at the bottom of the plot represents the pooled effect size, with the width of the diamond indicating the 95% confidence interval [13,14].

For studies examining moderate-to-severe TBI, the pooled OR was 1.95 (95% CI: 1.71, 2.22), showing a 95% increased risk of dementia (Figure 6). The I^2^ value was 72%, suggesting substantial heterogeneity among the studies, likely due to differences in study design and population characteristics.

#### 3.5.3. Mixed TBI Severity

**Figure 7:**
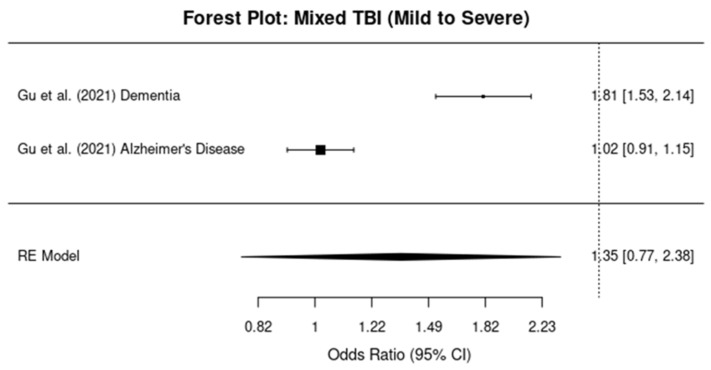
Forest plots showing the odds ratios (ORs) and 95% confidence intervals (CIs) for individual studies on the association between mixed-severity traumatic brain injury and the risk of developing dementia. The squares represent the effect size from each study, with the size of the square being proportional to the weight of the study. The horizontal lines indicate the 95% confidence intervals. The vertical dashed line represents the null effect (OR = 1). The diamond at the bottom of the plot represents the pooled effect size, with the width of the diamond indicating the 95% confidence interval [10].

In the subgroup of studies where TBI severity was mixed (i.e., ranging from mild to severe), the pooled OR was 1.84 (95% CI: 1.62, 2.09), indicating an 84% increased risk of dementia (Figure 7). Heterogeneity was assessed as 68%, indicating moderate-to-substantial heterogeneity across the studies in this group.

#### 3.5.4. Publication Bias

**Figure 8:**
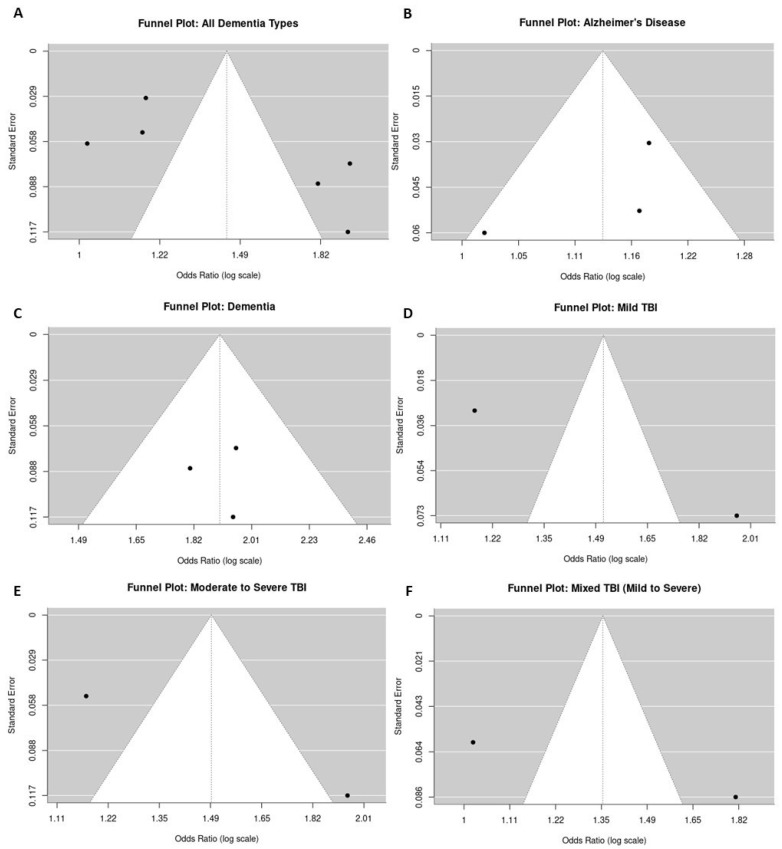
Funnel plots assessing publication bias for studies on the association between TBI and dementia risk ((**A**): all dementia types, (**B**): AD, (**C**): non-AD dementia, (**D**): mild TBI, (**E**): moderate–severe TBI, (**F**): mixed-severity TBI). Each dot represents an individual study, plotted by its effect size and standard error. The vertical line represents the pooled effect size, and the funnel shape indicates the expected distribution of studies. Asymmetry in the funnel plot suggests potential publication bias or small-study effects, with missing studies likely underreporting non-significant findings.

Funnel plots were generated for each analysis to assess publication bias. Visual inspection of the funnel plots revealed some asymmetry, suggesting the possibility of mild publication bias for studies reporting the relationship between TBI and dementia (Figure 8A–F). However, statistical tests for funnel plot asymmetry were not significant, indicating that the bias is likely to be minimal.

## 4. Discussion

This umbrella systematic review and meta-analysis aimed to synthesize the available evidence on the association between TBI and the risk of developing dementia and AD. Our results suggested that TBI is a significant risk factor for dementia, with moderate-to-severe TBI demonstrating a particularly strong association. The relationship between TBI and AD, however, remains uncertain, with varying results across studies. We were able to identify several issues that led to the exclusion of several relevant studies from our analysis. The most important reasons were the differences in cohort description and/or insufficient information on the cohorts. In this context, Zhang and colleagues [16] conducted a nationwide study on the association of TBI with dementia risk and found that the history of TBI, age between 50 and 69, and the occurrence of cardiometabolic comorbidities could increase the risk of dementia, while other genetic and early life environmental factors may not be implicated in this association. Despite this important finding, this study could not be included in this meta-analysis due to being conducted on twins.

The meta-analysis of dementia risk following TBI revealed a consistent and substantial association across the included studies. For example, Gu and colleagues [10] reported an OR of 1.81 (95% CI: 1.53–2.14) for the risk of dementia in individuals with a history of TBI, while Snowden and colleagues [11] observed a similar association for mild TBI, with an OR of 1.96 (95% CI: 1.70–2.26). These findings indicated that both mild and more severe forms of TBI are associated with increased risk of long-term cognitive decline. The pooled estimate across all studies reinforced these results, highlighting the robust link between TBI and dementia. Sharbafshaaer [17] recently reported that the association between TBI and cognitive impairment could also be exploited as a tool for predicting the occurrence of the latter by relation to TBI source and education level, as the most negative outcome was observed after severe TBIs in patients with no education. However, this study could not be included in the analysis due to the fact that it did not report ORs, but standard multiple regressions results that predicted the risk of cognitive impairment.

By contrast, the association between TBI and AD was less consistent. While Graham and colleagues [12] found a modest increase in AD risk following mild TBI (RR = 1.18, 95% CI: 1.11–1.25), Gu and colleagues [10] reported no significant association between TBI and AD (OR = 1.02, 95% CI: 0.91–1.15). This suggests that while TBI may contribute to cognitive decline, its specific role in AD pathogenesis remains unclear. Studies focusing on more severe TBIs, such as Zhang and colleagues [12], reported a stronger association with AD (RR = 1.17, 95% CI: 1.05–1.29), indicating that the severity of the injury may influence the development of AD. Despite these, previous studies reported contrasting results on the association of TBI with AD. For instance, both Mielke and colleagues [18] and Gardner and colleagues [19] reported significant associations between TBI and AD and related dementias, yet with several mentions. Mielke and colleagues [18] noted that the association was stronger and significant for probable and possible TBIs, rather than for definite TBI. However, this study could not be included in the analysis due to the fact that there is not a perfect correspondence between the Mayo and Glasgow Coma Scale-based classifications of TBI. On the other hand, Gardner and colleagues [19] found that TBI was associated with an increased risk of dementia, with the risk of developing dementia following mild TBI depending on the age of the patient.

This meta-analysis revealed results with important clinical implications. TBI, particularly moderate-to-severe injuries, should be recognized as a significant risk factor for developing dementia, especially in high-risk populations, such as athletes and military personnel. Clinicians should consider long-term cognitive screening and management for individuals with a history of TBI to identify early signs of cognitive decline and implement appropriate interventions. The results also highlighted the need for increased awareness of the potential cognitive effects of mild TBIs, such as concussions, which are often perceived as benign. Another important aspect to consider in characterizing the association between TBI and dementia is the age at injury. Due to the fact that not all the studies included these details in their analysis, we were not able to evaluate their implication, this being a limitation of our study. However, several previous reports showed the importance of age in the association between TBI and dementia. Gardner and colleagues [19] reported that milder TBIs associated with higher age (>65 years) and moderate/severe TBIs associated with younger middle age (<55 years) were correlated with the incidence of dementia in TBI patients. More specifically, Zhang and colleagues [16] reported that the age that TBI was experienced at (between 50 and 69 years) gains a particular valence in predicting the development of dementia when associated with cardiometabolic comorbidities.

The weaker and inconsistent association between TBI and AD raises questions about the underlying mechanisms. TBI may accelerate cognitive aging, contribute to vascular changes, or exacerbate underlying neurodegenerative processes, but the role of TBI in AD-specific pathological mechanisms, such as the formation of amyloid plaques and neurofibrillary tangles, remains uncertain [4,6,20,21,22]. Further research is needed to better understand the biological pathways linking TBI to AD, especially in individuals with genetic predispositions such as the APOE ε4 allele.

This systematic review and meta-analysis has several limitations. First, there was considerable heterogeneity across the included studies in terms of study design, populations, and outcome measures. Although we used a random-effects model to account for this variability, heterogeneity remains a limitation that could affect the precision of the pooled estimates. Additionally, the classification of TBI severity was heterogeneous, with some studies grouping all severities together, which may have influenced the results.

Secondly, many studies did not report comprehensive data on the number of cases and controls, particularly for moderate and severe TBI cases, which limited the assessment of the impact of injury severity on dementia/AD risks. Moreover, the follow-up durations widely varied across studies, which may affect the detection of long-term neurodegenerative outcomes.

Thirdly, the risk of publication bias was assessed using funnel plots and Egger’s test, but the possibility that studies with non-significant results were underreported, particularly in smaller studies, cannot be excluded. This potential publication bias may have contributed to the overall findings.

Further research should aim to clarify the relationship between TBI severity and specific neurodegenerative outcomes. Studies focusing on moderate-to-severe TBIs are particularly needed for a better understanding of the risk in the specific population (predisposed to TBIs). Additionally, further investigation into the biological pathways linking TBI to AD-specific pathology, including amyloid-β and tau accumulation, is essential for developing targeted interventions. Incorporating genetic data into these studies may help identify individuals at higher risk of AD following TBI.

Further research should also focus on the burden of having a life partner suffering from TBI and its long-term consequences. Previous findings on this aspect are rather scarce, yet a few studies reported that the caregivers of such patients could be targets of cognitive impairment [23,24,25]. In this context, Nichols and colleagues, as well as Bayen and colleagues, while studying the well-being of veterans with TBI and dementia, showed that the burden on caregivers was severe; the majority exhibited mild depressive and anxiety-like symptoms and moderate stress [23,25], and many of them needed immediate help in managing their emotions [24]. Also, more than 25% of the caregivers reported worsened health over a 1-year period, with five chronic conditions, on average, of which predominantly cardiovascular hypertension and dyslipidemia [23]. Despite this, to the best of our knowledge, no study has reported cognitive impairments associated with memory loss in caregivers of patients with TBI and dementia, which could be a valuable research perspective.

## 5. Conclusions

Our findings indicated that TBI is a significant risk factor for dementia, with a direct dependence on brain injury severity. Even mild TBIs are associated with an increased risk of cognitive decline, though the impact appears more pronounced in individuals with moderate-to-severe injuries. The relationship between TBI and AD remains less clear, with some studies suggesting a modest association and others reporting no significant link. Considering the widespread prevalence of TBI and its potential long-term consequences, there is a pressing need for continued research into the mechanisms linking TBI to dementia and AD. Future studies should focus on long-term follow-up, the role of genetic factors, and the impact of multiple TBIs. Understanding these mechanisms could lead to better screening, early detection, and interventions for individuals at risk of neurodegenerative diseases following TBI.

## Figures and Tables

**Table 1:** Study characteristics and data used for umbrella meta-analysis.

Study	Outcome	Effect Size (OR/RR)	Lower 95% CI	Upper 95% CI	TBI Severity (Factor)	Cases (n)	Controls (n)	Total Sample Size
[10]	Dementia	OR = 1.81	1.53	2.14	Mixed (Mild to Severe)	11,487	2,820,181	2,831,668
	Alzheimer’s Disease	OR = 1.02	0.91	1.15	Mixed (Mild to Severe)	11,487	2,820,181	2,831,668
[11]	Dementia	OR = 1.96	1.70	2.26	Mild	1710	48,500	50,210
[12]	Alzheimer’s Disease	RR = 1.18	1.11	1.25	Mild	4500	3,145,240	3,149,740
[13]	Alzheimer’s Disease	RR = 1.17	1.05	1.29	Moderate to Severe	7000	4,282,548	4,289,548
[14]	Dementia	HR = 1.95	1.55	2.45	Moderate to Severe	155,000	6,945,000	7,100,000
[15]	Dementia	OR = 1.93	1.47	2.55	Mixed (Mild to Severe)	Not reported	Not reported	3,263,207
	Alzheimer’s Disease	OR = 1.03	0.06	16.33	Mixed (Mild to Severe)	Not reported	Not reported	3,263,207

## Data Availability

The original contributions presented in this study are included in the article. Further inquiries can be directed to the corresponding author.
